# Silibinin suppresses glioblastoma cell growth, invasion, stemness, and glutamine metabolism by YY1/SLC1A5 pathway

**DOI:** 10.1515/tnsci-2022-0333

**Published:** 2024-02-24

**Authors:** Ming Liu, Xipeng Liu, Jianxin Qiao, Bing Cao

**Affiliations:** Department of Neurosurgery, The First Affiliated Hospital of Hebei North University, 12 Changqing Road, Qiaoxi District, Zhangjiakou City, 075000, Hebei Province, China

**Keywords:** glioblastoma, Silibinin, SLC1A5, YY1

## Abstract

**Background:**

Silibinin has been found to inhibit glioblastoma (GBM) progression. However, the underlying molecular mechanism by which Silibinin regulates GBM process remains unclear.

**Methods:**

GBM cell proliferation, apoptosis, invasion, and stemness are assessed by cell counting kit-8 assay, EdU assay, flow cytometry, transwell assay, and sphere formation assay. Western blot is used to measure the protein expression levels of apoptosis-related markers, solute carrier family 1 member 5 (SLC1A5), and Yin Yang-1 (YY1). Glutamine consumption, glutamate production, and α-ketoglutarate production are detected to evaluate glutamine metabolism in cells. Also, SLC1A5 and YY1 mRNA levels are examined using quantitative real-time PCR. Chromatin immunoprecipitation assay and dual-luciferase reporter assay are used to detect the interaction between YY1 and SLC1A5. Mice xenograft models are constructed to explore Silibinin roles *in vivo*.

**Results:**

Silibinin inhibits GBM cell proliferation, invasion, stemness, and glutamine metabolism, while promotes apoptosis. SLC1A5 is upregulated in GBM and its expression is decreased by Silibinin. SLC1A5 overexpression abolishes the anti-tumor effect of Silibinin in GBM cells. Transcription factor YY1 binds to SLC1A5 promoter region to induce SLC1A5 expression, and the inhibition effect of YY1 knockdown on GBM cell growth, invasion, stemness, and glutamine metabolism can be reversed by SLC1A5 overexpression. In addition, Silibinin reduces GBM tumor growth by regulating YY1/SLC1A5 pathway.

**Conclusion:**

Silibinin plays an anti-tumor role in GBM process, which may be achieved via inhibiting YY1/SLC1A5 pathway.

## 1 Introduction

Glioblastoma (GBM) is a glioma with the highest degree of malignancy among astrocyte tumors, belonging to WHO grade IV, and the disease progresses rapidly [1]. About 90% of GBM patients have a local recurrence within 2 years after treatment, and there is no effective treatment for patients who have recurrence [2]. Huge inter- and intra-tumoral heterogeneity is the main manifestation of GBM, thus making it more difficult to treat [3]. Therefore, exploring the molecular mechanisms that influence GBM processes is critical in developing potential molecular targets for GBM therapy.

Silibinin is a major flavonoid lignan isolated from the fruit or seeds of milk thistle and has long been used as an antioxidant and hepatoprotective agent [4,5]. Silibinin has been reported to play vital function in a variety of diseases. Song et al. reports that Silibinin has a variety of pharmacological effects on hepatobiliary diseases such as hepatitis and cirrhosis [6]. Wei et al. reveal that Silibinin has a positive effect in the treatment of Alzheimer’s disease [7]. Previous study suggested that Silibinin played anti-tumor role in GBM, whose treatment inhibited the metabolic activity, proliferation, and invasive features of GBM cells [8–10]. However, the molecular mechanism of Silibinin in GBM progression remains unclear.

Glutamine is an essential nutrient for cancer cell signaling and regulates energy production and redox balance [11,12]. Solute carrier family 1 member 5 (SLC1A5) is a transporter of mitochondrial glutamine for cancer metabolic reprogramming [13]. SLC1A5 has been found to be associated with eight types of tumor immune-infiltrating cells and immune state, suggesting that it can be used as a pharmacological target for the development of novel anti-cancer drugs [14]. Furthermore, Han et al. identified SLC1A5 as a prognostic biomarker and potential therapeutic target for gliomas [15]. In this, we found that Silibinin could reduce the expression of SLC1A5 in GBM cells. However, it is unclear whether Silibinin mediates GBM processes by regulating SLC1A5.

Yin Yang-1 (YY1) is an oncogenic transcription factor that regulates enhancer–promoter junctions [16]. Various findings have demonstrated that YY1 is upregulated in many tumors and is associated with the poor outcome of patients [17,18]. Importantly, YY1 expression was elevated in GBM, and it could promote GBM cell proliferation and metastasis to accelerate malignancy progression [19,20]. Moreover, YY1 could bind to SENP1 promoter region to enhance its transcriptional expression, thereby mediating self-renewal of GBM-stem cells [21]. Here, we used the jaspar software to predict that YY1 had binding sites with SLC1A5 promoters. Nevertheless, whether YY1 mediates GBM processes by binding to SLC1A5 is unclear.

This study aimed to explore the mechanism of Silibinin in the progression of GBM. Through our analyses, we proposed the following hypotheses and verified that Silibinin affects the malignant progression of GBM by mediating the YY1/SLC1A5 pathway.

## 2 Materials and methods

### 2.1 Bioinformatics data analysis

The SLC1A5/YY1 mRNA expression data in 163 patients with GBM and 207 normal brain tissues were downloaded from The Cancer Genome Atlas (TCGA) database (http://cancergenome.nih.gov/). Gene expression profiling interactive analysis (GEPIA) database (http://gepia.cancer-pku.cn/detail.php) was used to analyze SLC1A5/YY1 mRNA expression in 156 GBM tissues and 5 normal brain tissues. Besides, the clinical data of SLC1A5/YY1 protein expression in 99 primary GBM patients and 10 normal brain tissues were procured from Clinical Proteomic Tumor Analysis Consortium (CPTAC) database (https://ualcan.path.uab.edu/analysis-prot.html).

### 2.2 Samples

After surgical resection, tissue samples were collected from 31 GBM patients recruited at The First Affiliated Hospital of Hebei North University between July 2019 and July 2021. Inclusion criteria were diagnosed as GBM through histopathological analysis, not treated with surgical resection, chemotherapy or radiation therapy prior to admission, and received surgical resection of the primary tumors after admission. Exclusion criteria included: patients with mental disorders, infectious diseases, other cancers or a history of treatment for other cancers. Meanwhile, normal brain tissues from 31 traumatic brain injury patients (the body health without any other disease before injured) as negative controls at this hospital. Experienced pathologists identified GBM tissue samples according to the diagnostic standards of World Health Organization (WHO). Samples were stored at –80°C. Each participant signed written informed consent. The relationships between SLC1A5/YY1 expression and clinicopathologic features of GBM patients are listed in Tables 1 and 2.

**Table 1:** Relationship between SLC1A5 expression and clinicopathologic features of glioblastoma patients

	Characteristics (*n* = 31)	SLC1A5 expression		*P* value
		Low (*n* = 15)	High (*n* = 16)	
Gender				0.7244
Female	15	8	7	
Male	16	7	9	
WHO grade				0.0320*
I + II	14	10	4	
III + IV	17	5	12	
Tumor size				0.0038*
≤3 cm	14	11	3	
>3 cm	17	4	13	

*P < 0.05.

**Table 2:** Relationship between YY1 expression and clinicopathologic features of glioblastoma patients

	Characteristics (*n* = 31)	YY1 expression		*P* value
		Low (*n* = 15)	High (*n* = 16)	
Gender				>0.9999
Female	15	7	8	
Male	16	8	8	
WHO grade				0.0054*
I + II	14	11	3	
III + IV	17	4	13	
Tumor size				0.0210*
≤3 cm	14	10	4	
>3 cm	17	5	12	

*P < 0.05.

### 2.3 Cell culture and treatment

Human normal astrocytes (NHA) and GBM cells (A172 and U251) (all from Procell, Wuhan, China) were cultured in DMEM containing 10% FBS and 1% penicillin/streptomycin (Gibco, Grand Island, NJ, USA) at 37°C with 5% CO_2_. To explore the effect of Silibinin on GBM cells, A172 and U251 cells were treated with Silibinin (Sigma-Aldrich, St Louis, MO, USA) for 48 h.

### 2.4 Cell transfection

The pcDNA overexpression vector for SLC1A5 and YY1, siRNA against YY1 (si-YY1: F 5′-GGUCAUAGAUGCAGAAAUAUA-3′, R 5′-UAUUUCUGCAUCUAUGACCUA-3′), and negative control (pcDNA, si-NC: F 5′-UUCUCCGAACGUGUCACGUTT-3′, R 5′-ACGUGACACGUUCGGAGAATT-3′) (RiboBio, Guangzhou, China) were transfected into GBM cells using Lipofectamine 3000 (Invitrogen, Carlsbad, CA, USA). After that, transfected cells were treated with 150 μM Silibinin for 48 h.

### 2.5 Cell counting kit-8 (CCK8) assay

GBM cells were seeded into 96-well plates (5 × 10^3^ cells/well) and cultured for 48 h. Then, cells were treated with 10 μL CCK8 reagent (Dojindo, Kumamoto, Japan) for 2 h. Cell viability was determined at 450 nm by a microplate reader (Epoch, BioTek, Winooski, Vermont, USA).

### 2.6 EdU assay

GBM cells were seeded into 96-well plates (1 × 10^4^ cells/well) followed by labeling with EdU solution and stained with DAPI based on EdU Kit (RiboBio) instructions. EdU positive cells were observed using fluorescence microscopy (SMZ18, Nikon, Tokyo, Japan).

### 2.7 Flow cytometry

After treatment or transfection, GBM cells were harvested into a tube (5 × 10^5^ cells). Then, cells were washed with PBS, suspended with binding buffer, and colored with Annexin V-FITC and PI solution (Beyotime, Shanghai, China). Following this, cell apoptosis was analyzed by flow cytometer (LSRFortessa TM X-20, BD Biosciences, San Diego, CA, USA).

### 2.8 Transwell assay

Matrigel-coated Transwell chambers (Corning Inc., Corning, NY, USA) were seeded with GBM cells (4 × 10^5^ cells/well) suspended in serum-free medium, and the lower chamber was added with complete medium. Then, the invaded cells were counted under a microscope (E100, Nikon) after being fixed using paraformaldehyde and stained by crystal violet.

### 2.9 Sphere formation assay

Cells were plated in ultra-low attachment 24-well plates (500 cells/well) with serum-free medium containing 20 ng/mL basic fibroblast growth factor and 20 ng/mL epidermal growth factor. Then, sphere formation efficiency was counted using a microscope (E100, Nikon).

### 2.10 Western blot (WB) analysis

Proteins were extracted with RIPA buffer, uploaded on 10% SDS-PAGE gel, and transferred to PVDF membrane. Membrane was blocked with 5% non-fat milk for 2 h, and then incubated with antibodies (Abcam, Cambridge, CA, USA), including anti-SLC1A5 (1:1,000, ab237704), anti-Bcl-2 (1:1,000, ab32124), anti-Bax (1:1,000, ab32503), anti-YY1 (1:1,000, ab109228), anti-Nanog (1:200, ab21624), anti-Sox2 (1:1,000, ab97959), anti-GAPDH (1:2,500, ab9485), and secondary antibody (1:50,000, ab205718). ECL reagent (Beyotime) was then used for visualizing protein bands, and gray value was analyzed by Image J software.

### 2.11 Detection of glutamine metabolism

According to the manufacturer’s instructions, Glutamate Assay Kit, Glutamine Assay Kit, and α-ketoglutarate Assay Kit (all from Abcam) were utilized to measure glutamate production, glutamine consumption, and α-ketoglutarate production in cell culture medium, respectively.

### 2.12 Quantitative real-time PCR (qRT-PCR)

Total RNAs were isolated by TRIzol reagent (Invitrogen) and reverse-transcribed by cDNA Synthesis Kit (Takara, Tokyo, Japan). PCR amplification was carried out using SYBR Green (Takara) with specific primers (Table 3) under following thermocycling conditions: 95°C for 3 min, 40 cycles of 95°C for 20 s, 60°C for 30 s, and 72°C for 30 s. Relative mRNA expression was analyzed using 2^−ΔΔCt^ method with GAPDH as internal control.

**Table 3:** Primer sequences used for qRT-PCR

Name		Primers for PCR (5′–3′)
SLC1A5	Forward	CTCCAGCCCTCGGGAGTAAATA
	Reverse	GGCGTACCACATGATCCAGG
YY1	Forward	AAGCTGCACTTTCTTGGGGT
	Reverse	ACCATCTTCAGGCAACCAGG
GAPDH	Forward	GACCACAGTCCATGCCATCAC
	Reverse	ACGCCTGCTTCACCACCTT

### 2.13 Chromatin immunoprecipitation (ChIP) assay

Using EZ-ChIP Kit (Millipore, Billerica, MA, USA), GBM cells were transfected with pcDNA binding site 1/2 and then incubated with formaldehyde. After cross-linked chromatins were sonicated into fragments, fragments were immunoprecipitated with anti-YY1 or anti-IgG. The precipitated DNA was eluted and examined by qRT-PCR (Site 1: F 5′-AGCTGGGACTACAAGTGCAT-3′, R 5′-AGTGAGACCTTATCTTTAAAAACATGA-3′; Site 2: F 5′-ACATTACCACTACAATCTTTGAGCA-3′, R 5′-CATGCCCACCTCCCAAGC-3′).

### 2.14 Dual-luciferase reporter assay

The SLC1A5 promoter regions containing YY1 binding sites and mutated sites were introduced into the pGL3-basic vector to generate wild-type (WT)/mutant-type (MUT)-SLC1A5 vectors. 293T cells in 24-well plates were co-transfected with si-NC/si-YY1 and WT/MUT-SLC1A5 vectors. After 48 h, the luciferase activity was analyzed by corresponding Assay Kit (Beyotime).

### 2.15 Mice xenograft models

U251 cells were injected into the right flank of BALB/c nude mice (Vital River, Beijing, China). After 7 days, Silibinin (60 mg/kg) was administered into mice every 3 days with PBS injection used as control. The tumor volume was measured with a vernier caliper every 3 days after 7 days according to the formula: tumor volume  =  (width^2^ × length)/2. After 22 days, tumor tissues were collected. Additionally, tumor tissues were used to prepare paraffin sections for conducting immunohistochemical (IHC) staining with anti-SLC1A5 (1:100, ab237704), anti-YY1 (1:250, ab109228), and anti-PCNA (1:250, ab18197). Animal study was approved by the Animal Ethics Committee of The First Affiliated Hospital of Hebei North University.

### 2.16 Statistical analysis

All data are shown as mean  ±  SD. Statistical significance was assessed by Student’s *t-*tests or ANOVA using GraphPad Prism 7.0 software. *P* < 0.05 was considered to denote statistical significance.


**Ethical approval:** The research related to human use has been complied with all the relevant national regulations, institutional policies and in accordance the tenets of the Helsinki Declaration, and has been approved by the authors’ institutional review board or equivalent committee. This study was approved by the Ethics Committee of The First Affiliated Hospital of Hebei North University.
**Informed consent:** Informed consent has been obtained from all individuals included in this study.

## 3 Results

### 3.1 Silibinin inhibits malignant progression of GBM

**Figure 1:**
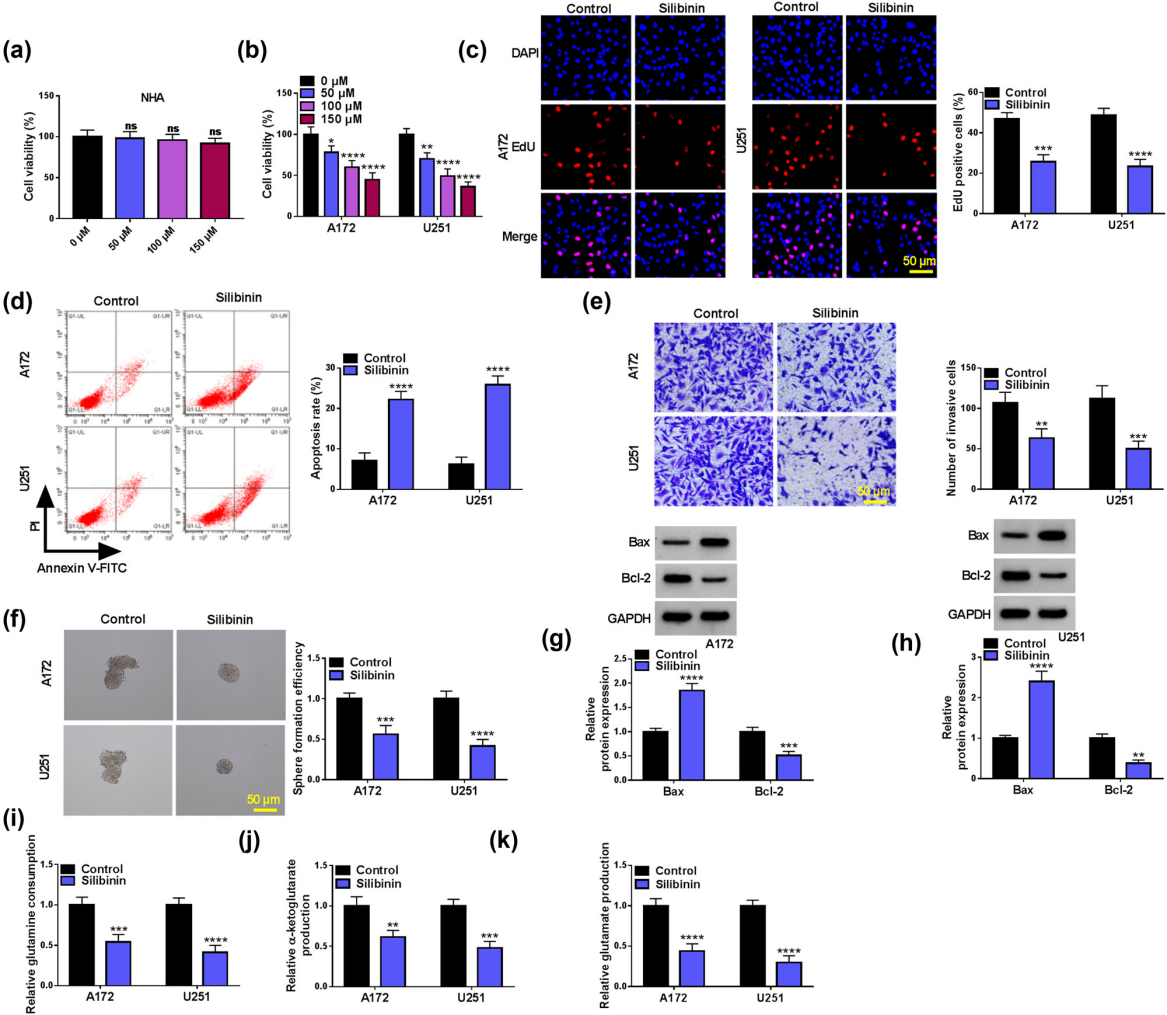
Effect of Silibinin on the malignant progression of GBM. (a and b) Viability of A172, U251, and NHA cells was detected by CCK8 assay under different concentrations of Silibinin. (c–k) A172 and U251 cells were treated with or without Silibinin. EdU assay (c), flow cytometry (d), transwell assay (e), and sphere formation assay (f) were used to detect cell proliferation, apoptosis, invasion, and stemness. (g and h) Detection of cellular Bax and Bcl-2 protein expression by WB. (i–k) Glutamine metabolism was assessed by detecting glutamine consumption, glutamate production, and α-ketoglutarate production. *P < 0.05, **P < 0.01, ***P < 0.001, ****P < 0.0001.

Under the treatment of different concentrations of Silibinin, the viability of normal astrocytes (NHA) has not changed, while the viability of GBM cells (A172 and U251) decreased with the increase of Silibinin concentration (Figure 1a and b). Through evaluation, 150 μM Silibinin is used for follow-up study. Our data show that Silibinin treatment inhibits the EdU positive cell rate, number of invaded cells, and sphere formation efficiency, while increasing the apoptosis rate in GBM cells (Figure 1c–f). By detecting the protein levels of stemness markers, Silibinin markedly reduces the protein levels of Nanog and Sox2 in A172 and U251 cells (Figure S1a and b). Furthermore, we examine the expression of apoptosis-related proteins, and discover that Silibinin treatment increases the expression of pro-apoptotic protein Bax and decreases the expression of anti-apoptotic protein Bcl-2 (Figure 1g and h). In addition, Silibinin reduces the glutamine consumption, glutamate production, and α-ketoglutarate production in GBM cells (Figure 1i–k). Thus, Silibinin restrains GBM cell growth, invasion, stemness, and glutamine metabolism.

### 3.2 SLC1A5 is upregulated in GBM and its expression is decreased by Silibinin

**Figure 2:**
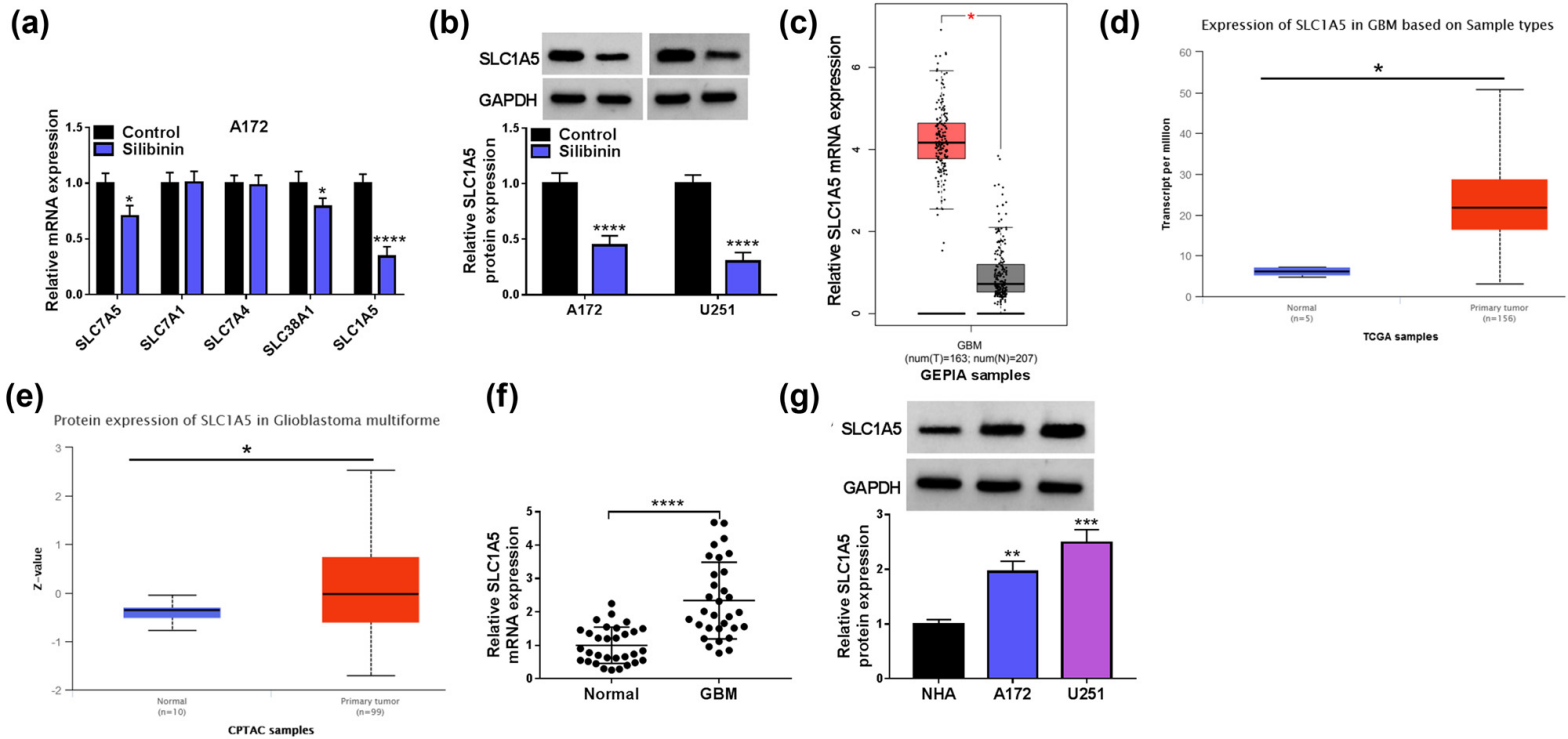
SLC1A5 expression in GBM and Silibinin-treated cells. (a) qRT-PCR for detection of SLC7A5, SLC7A1, SLC7A4, SLC38A1, and SLC1A5 mRNA expression in GBM cells treated with or without Silibinin. (b) Protein expression of SLC1A5 was detected by WB analysis. (c and d) GEPIA and TCGA databases analyzed the SLC1A5 mRNA expression in GBM tissues and normal tissues. (e) CPTAC database analyzed SLC1A5 protein levels in GBM tissues and normal tissues. (f) qRT-PCR was used to detect the expression of SLC1A5 in human normal tissues and GBM tissues. (g) SLC1A5 protein expression in NHA and GBM cells was assayed using WB. *P < 0.05, **P < 0.01, ***P < 0.001, ****P < 0.0001.

Then, we assess the effects of Silibinin on multiple glutamine transporters (SLC7A5, SLC7A1, SLC7A4, SLC38A1, and SLC1A5), and found that Silibinin has the strongest inhibitory effect on SLC1A5 mRNA expression (Figure 2a). Therefore, SLC1A5 is selected as a target of Silibinin for our study. Further analysis reveals that Silibinin treatment significantly inhibits the protein expression level of SLC1A5 in GBM cells (Figure 2b). In GBM tissues, GEPIA and TCGA database analyses have shown that SLC1A5 mRNA expression is upregulated (Figure 2c and d), and CPTAC database suggests that SLC1A5 protein level is significantly enhanced (Figure 2e). Additionally, SLC1A5 mRNA level is highly expressed in human GBM tissues and its protein level is also increased in GBM cells (Figure 2f and g). Besides, high SLC1A5 expression is correlated with the WHO grade and tumor size of GBM patients (Table 1). This evidence suggests that SLC1A5 has elevated expression in GBM, and Silibinin inhibits SLC1A5 expression.

### 3.3 Silibinin suppresses SLC1A5 expression to repress GBM progression

**Figure 3:**
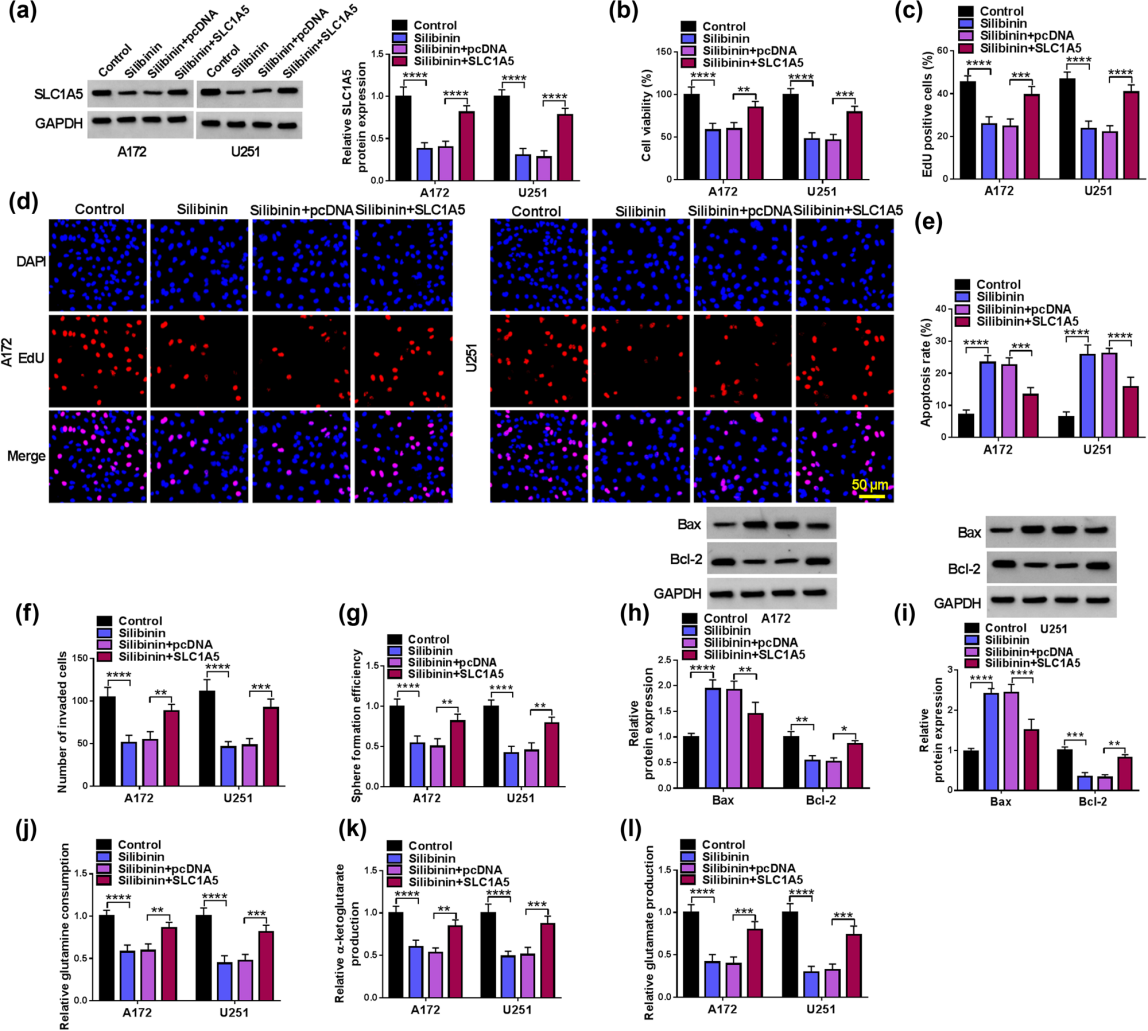
Effect of Silibinin and SLC1A5 on GBM progression. A172 and U251 cells were transfected with pcDNA/SLC1A5 overexpression vector and treated with Silibinin. (a) SLC1A5 protein expression was detected by WB. Cell proliferation, apoptosis, invasion, and stemness were determined using CCK8 assay (b), EdU assay (c and d), flow cytometry (e), transwell assay (f), and sphere formation assay (g). (h and i) Protein expression was evaluated by WB analysis. (j–l) Glutamine consumption, glutamate production, and α-ketoglutarate production were tested to assess glutamine metabolism. *P < 0.05, **P < 0.01, ***P < 0.001, ****P < 0.0001.

We detected the effect of SLC1A5 overexpression on GBM cell functions, and the results reveal that SLC1A5 overexpression promotes GBM cell viability, EdU positive cell rate, the number of invaded cells, sphere formation efficiency, glutamine consumption, glutamate production, and α-ketoglutarate production (Figure S2a–g). To determine whether Silibinin regulates SLC1A5 expression to mediate GBM progression, A172 and U251 cells are transfected with pcDNA/SLC1A5 overexpression vector and then treated with Silibinin. WB assay reveals that SLC1A5 overexpression vector significantly increases SLC1A5 protein expression in Silibinin-treated GBM cells (Figure 3a). SLC1A5 overexpression increases cell viability, EdU positive cell rate, invaded cell number, sphere formation efficiency, and Bcl-2 expression, while inhibits apoptosis rate and Bax expression in Silibinin-treated GBM cells (Figure 3b–i). The inhibiting effect of Silibinin on glutamine metabolism-related indexes can be abolished by SLC1A5 overexpression (Figure 3j–l). In summary, Silibinin represses GBM progression by inhibiting SLC1A5 expression.

### 3.4 Transcription factor YY1 induces SLC1A5 transcription

**Figure 4:**
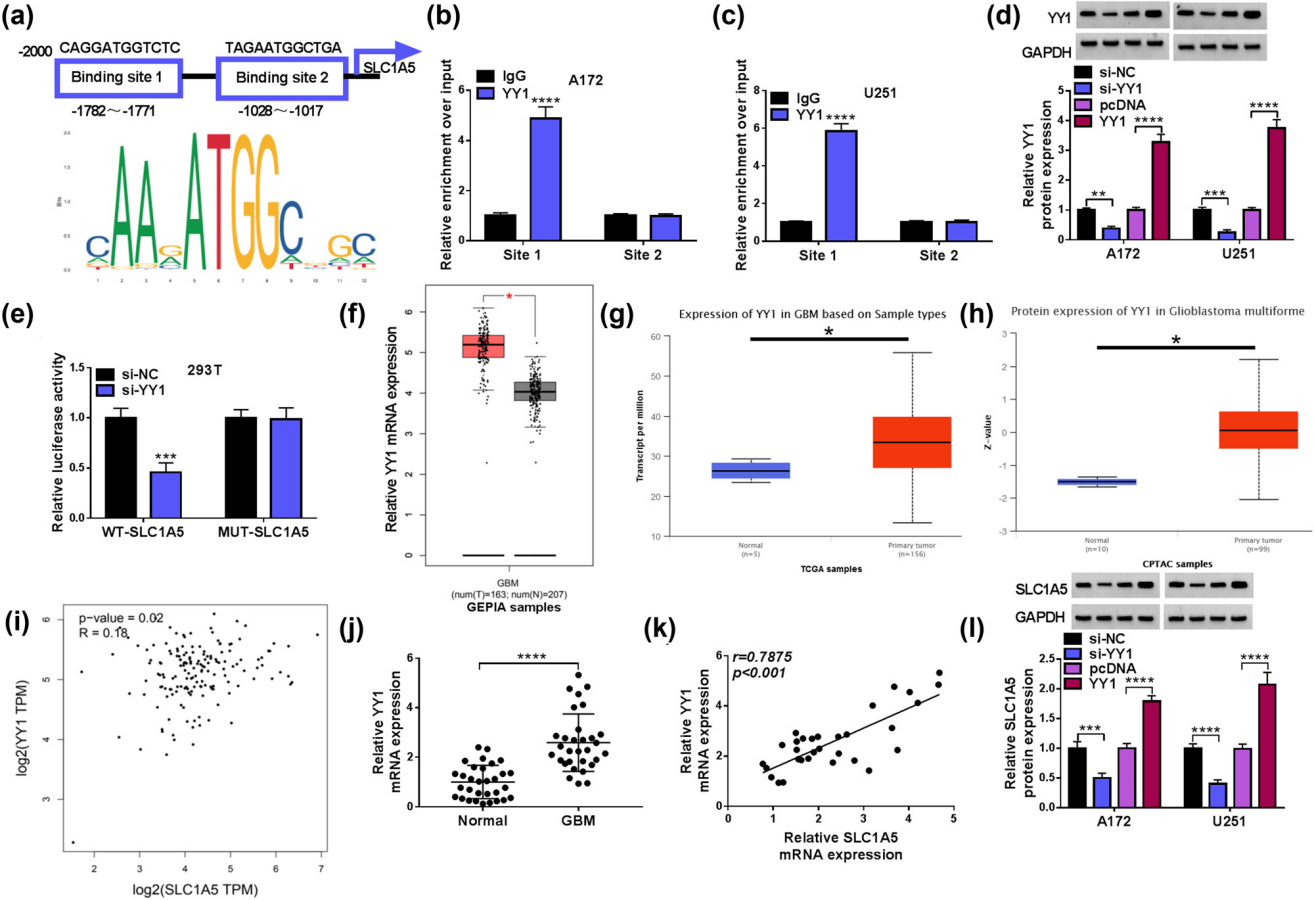
Interaction of YY1 and SLC1A5. (a) Jaspar was used to predict the binding sites of YY1 and SLC1A5. (b and c) ChIP was used to detect the interaction between YY1 and SLC1A5 binding sites. (d) Transfection efficiencies of si-YY1 and YY1 overexpression vector were confirmed by WB analysis. (e) Dual-luciferase reporter assay was used to detect the interaction between YY1 and SLC1A5. (f and g) GEPIA and TCGA databases analyzed YY1 mRNA expression in GBM tissues and normal tissues. (h) CPTAC database analyzed YY1 protein expression in GBM tissues and normal tissues. (i) GEPIA database analyzed the relationship between YY1 and SLC1A5 expression in GBM tissues. (j) qRT-PCR was used to detect YY1 mRNA expression in human normal tissues and GBM tissues. (k) Pearson correlation analysis was used to analyze the correlation between YY1 and SLC1A5 expression in GBM tissues. (l) SLC1A5 protein expression in GBM cells transfected with si-NC/si-YY1/pcDNA/YY1 was tested by WB. *P < 0.05, **P < 0.01, ***P < 0.001, ****P < 0.0001.

The jaspar software predicts that they have two binding sites between the transcription factor YY1 and the SLC1A5 promoter site (Figure 4a). ChIP analysis shows that SLC1A5 binding site 1 is significantly enriched in YY1 antibody (Figure 4b and c), suggesting that YY1 can interact with SLC1A5 promoter binding site 1 in GBM cells. Then, we construct si-YY1 and YY1 overexpression vector to decrease and increase YY1 protein expression in GBM cells, respectively (Figure 4d). Dual-luciferase reporter assay shows that si-YY1 inhibits the luciferase activity of WT-SLC1A5 vector (Figure 4e). Through the analysis of GEPIA, TCGA, and CPTAC databases, SLC1A5 was found to be highly expressed in GBM tissues at the mRNA and protein levels (Figure 4f–h). Moreover, GEPIA database reveals that YY1 expression is positively correlated with SLC1A5 expression in GBM tissues (Figure 4i). Detecting YY1 expression by qRT-PCR, YY1 mRNA expression is significantly higher in GBM tissues, and Pearson correlation analysis suggests that they have positive correlation between YY1 expression and SLC1A5 expression (Figure 4j and k). Through analysis, we confirm that YY1 expression is related to the WHO grade and tumor size of GBM patients (Table 2). Besides, SLC1A5 protein expression is decreased by YY1 knockdown and increased by YY1 overexpression in GBM cells (Figure 4l). The above concludes that YY1 can bind to SLC1A5 promoter to enhance its expression.

### 3.5 YY1 affects biological processes in GBM cells by regulating SLC1A5 expression

**Figure 5:**
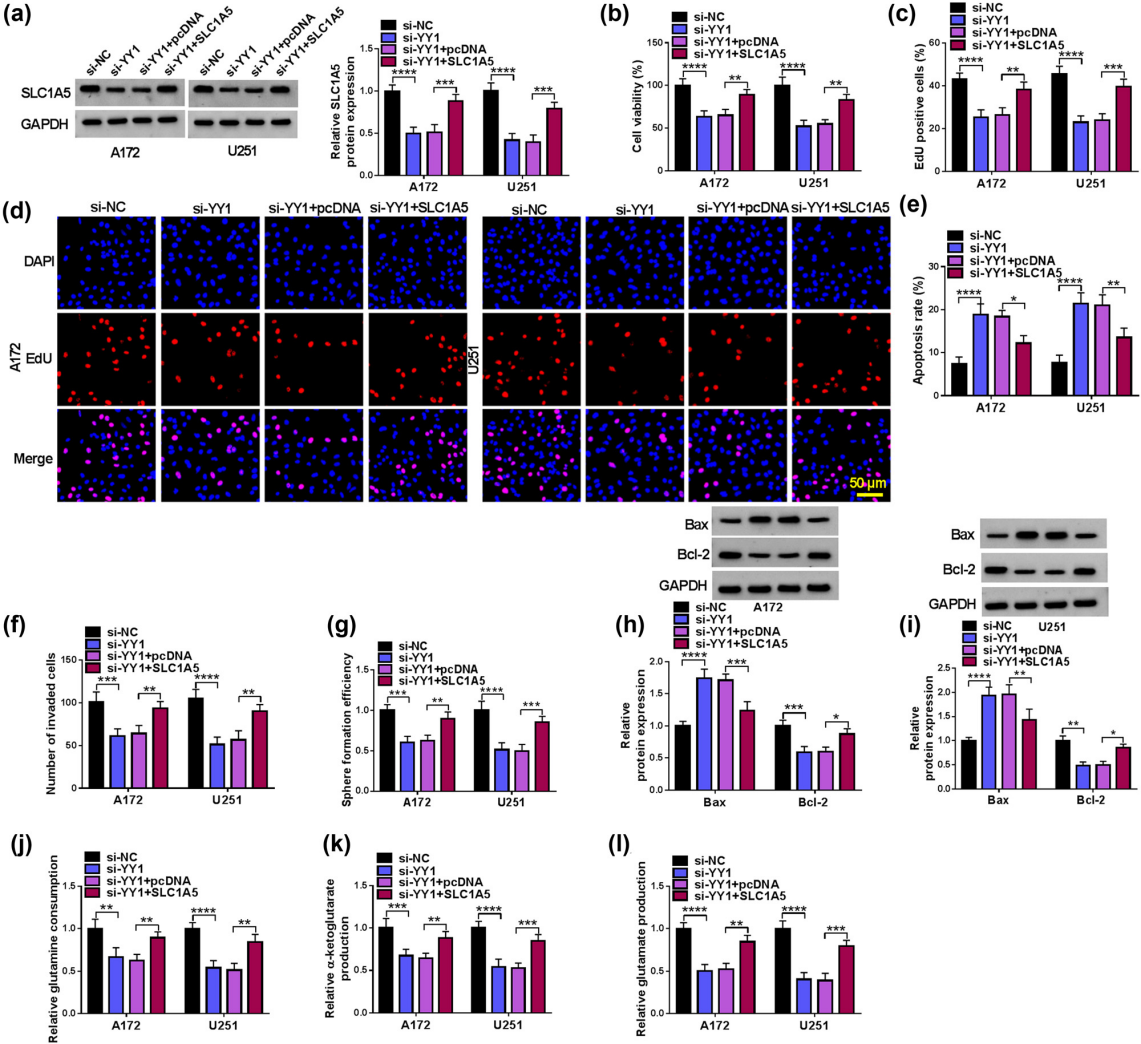
Effect of si-YY1 and SLC1A5 on biological processes in GBM cells. A172 and U251 cells were transfected with si-NC/si-YY1/pcDNA/SLC1A5 overexpression vector. (a) WB for detecting SLC1A5 protein expression. CCK8 assay (b), EdU assay (c and d), flow cytometry (e), transwell assay (f), and sphere formation assay (g) were performed to measure cell proliferation, apoptosis, invasion, and stemness. (h and i) Protein expression was examined by WB. (j–l) Glutamine metabolism was evaluated by testing glutamine consumption, glutamate production, and α-ketoglutarate production. *P < 0.05, **P < 0.01, ***P < 0.001, ****P < 0.0001.

To determine whether YY1 regulates GBM cell progression by influencing the expression of SLC1A5, GBM cells are transfected with si-YY1 and SLC1A5 overexpression vector. It is found that SLC1A5 protein expression suppressed by si-YY1 can be remarkably increased by SLC1A5 overexpression vector in GBM cells (Figure 5a). YY1 knockdown represses cell viability, EdU positive cell rate, invaded cell number, sphere formation efficiency, and Bcl-2 expression, while promotes apoptosis rate and Bax expression in GBM cells. However, these effects are reversed by SLC1A5 overexpression (Figure 5b–i). Downregulation of YY1 also reduces the glutamine consumption, glutamate production, and α-ketoglutarate production in GBM cells, while SLC1A5 overexpression overturns these effects (Figure 5j–l). Thus, YY1 may promote GBM progression by increasing SLC1A5 expression.

### 3.6 Silibinin mediates SLC1A5 expression by affecting YY1

**Figure 6:**
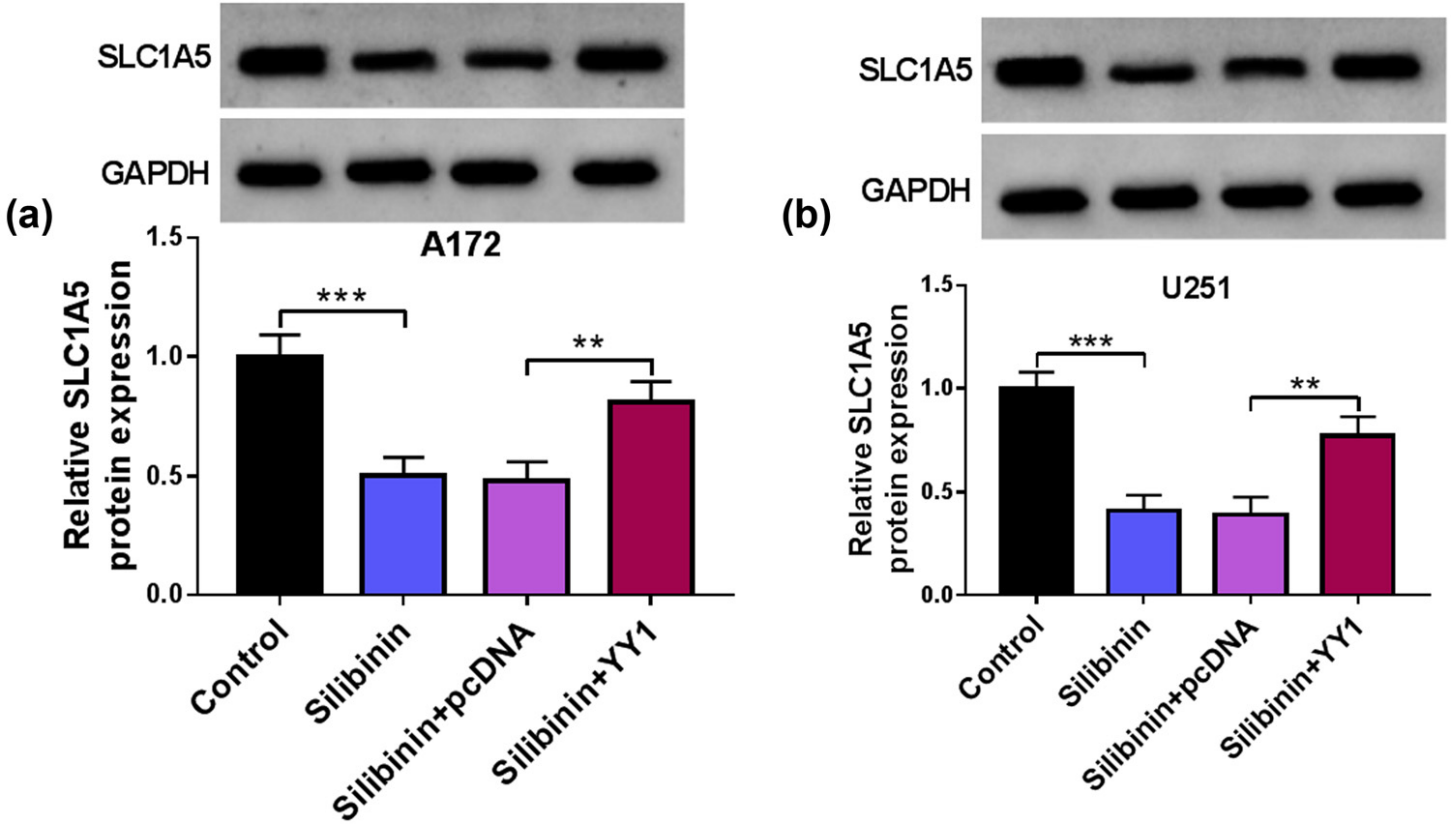
Effect of Silibinin and YY1 on SLC1A5 expression. SLC1A5 protein expression was measured by WB in A172 cell (a) and U251 cell (b) transfected with pcDNA/YY1 overexpression vector and treated with Silibinin. **P < 0.01, ***P < 0.001.

To detect the effect of Silibinin on the YY1/SLC1A5 pathway, GBM cells are transfected with pcDNA/YY1 overexpression vector and then treated with Silibinin. The detection of SLC1A5 protein expression results reveal that YY1 overexpression can reverse the decreasing effect of Silibinin on SLC1A5 expression (Figure 6a and b), suggesting that Silibinin reduces SLC1A5 expression by affecting YY1.

### 3.7 Silibinin restrains GBM tumor growth *in vivo*

**Figure 7:**
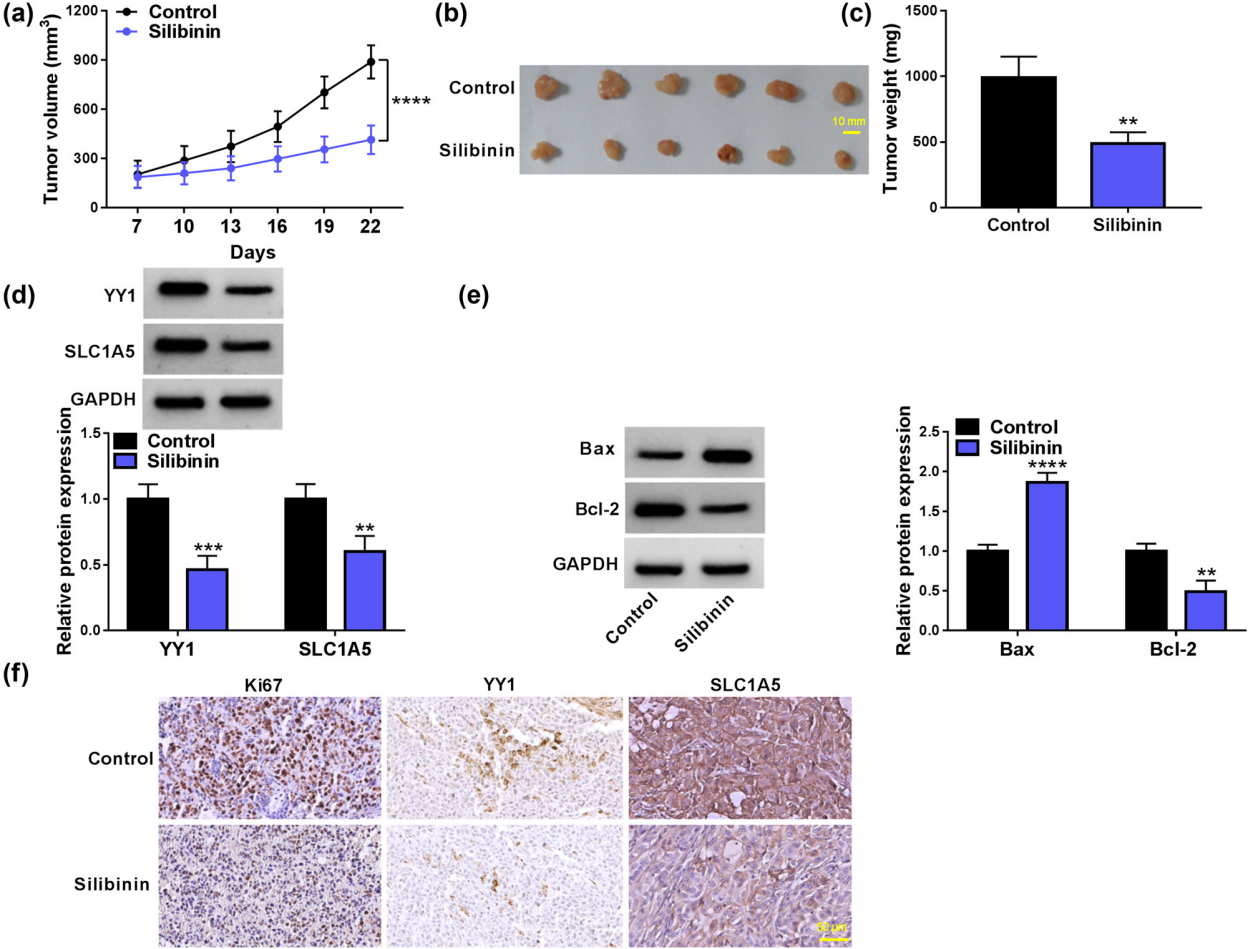
Effect of Silibinin on GBM tumor growth *in vivo*. (a) Tumor volume was measured every 3 days after 7 days. (b and c) Tumor weight was examined after 22 days. (d and e) Protein expression of YY1, SLC1A5, Bax, and Bcl-2 in mice tumor tissues was detected by WB analysis. (f) IHC staining showed the Ki67, YY1, and SLC1A5 positive cells in mice tumor tissues. **P < 0.01, ***P < 0.001, ****P < 0.0001.

Further analysis is performed to explore the role of Silibinin *in vivo*. Silibinin treatment significantly inhibits tumor volume and weight (Figure 7a–c). Besides, YY1, SLC1A5, and Bcl-2 protein levels are significantly reduced, while Bax protein level is markedly enhanced in the tumor tissues of Silibinin-treated group (Figure 7d and e). Moreover, IHC staining shows a significant reduction in the number of Ki67, YY1, and SLC1A5-positive cells in the tumor tissues of the Silibinin-treated group (Figure 7f). Taken together, these results suggest that Silibinin represses GBM tumor growth by inhibiting the YY1/SLC1A5 pathway.

## 4 Discussion

At present, the therapeutic effect of GBM is limited by tumor invasion and drug/radiation resistance. Therefore, it is of major significance to identify targeted therapeutic agents for the GBM patients. Many natural herbs in nature have anti-cancer activity and can inhibit the growth, invasion, as well as stemness of tumor cells [22]. For instance, dietary flavonoids has been confirmed to overcome GBM resistance and can serve as a potential alternative therapy for GBM [23]. More and more people are beginning to focus on the mechanism of Silibinin in anti-cancer therapies, such as lung cancer [24] and ovarian cancer [25]. Importantly, the role of Silibinin in GBM treatment has been reported [26]. In our study, Silibinin was found to inhibit GBM cell growth, invasion, stemness, and glutamine metabolism, as well as reduce GBM tumor growth *in vivo*. These results further confirmed the potential of Silibinin for GBM treatment.

Glutamine metabolism can be rewritten during tumorigenesis and can act as a pharmacological target for many cancers [27,28]. As a glutamine transporter, SLC1A5 has been demonstrated to have an essential role in glutamine metabolism [13,29]. A growing number of studies report that high SLC1A5 expression is associated with poor prognosis in cancer [30]. Although there is evidence that SLC1A5 is upregulated in GBM [31], its specific role in GBM process remains to be further revealed. In this, SLC1A5 was found to be reduced by Silibinin, and its overexpression also reversed the suppressing effect of Silibinin on GBM cell growth, invasion, stemness, and glutamine metabolism. These results confirmed that SLC1A5 acted as a tumor-promoting factor in GBM, and Silibinin affected GBM process by inhibiting glutamine metabolism through reducing SLC1A5 expression.

YY1 is a zinc finger protein that can activate or inactivate gene expression depending on the interacting promoter context [32]. YY1 enhanced the aerobic glycolysis and proliferation of neuroblastoma cell via increasing Lactate dehydrogenase A (LDHA) expression by binding to the promoter of LDHA [33]. Besides, LINC00858 facilitated gastric cancer cell metastasis, which could be regulated by transcription factor YY1 [34]. In this, we found that YY1 could bind to SLC1A5 promoter to increase its expression. The pro-tumor effect of YY1 in GBM process has been confirmed [19,20]. Consistent with these data, our data showed that silencing of YY1 restrained GBM cell growth, invasion, stemness, and glutamine metabolism. Moreover, SLC1A5 overexpression reversed the regulation of YY1 knockdown on GBM cell progression, verifying that YY1 promoted GBM progression by increasing SLC1A5 expression. More importantly, YY1 expression was reduced by Silibinin *in vivo*, and its overexpression enhanced SLC1A5 expression in Silibinin-induced GBM cells, confirming that Silibinin suppressed SLC1A5 expression by regulating YY1.

In conclusion, our findings provided a new direction for GBM treatment. Our data confirmed that Silibinin suppressed growth, invasion, stemness, and glutamine metabolism in GBM cells by mediating the YY1/SLC1A5 pathway. By uncovering the molecular mechanism of Silibinin in GBM, our study offers new evidence for the development of molecular targets for GBM therapy.

## Supplementary Material

Supplementary Figure
