# Supplementary material for: Silibinin suppresses glioblastoma cell growth, invasion, stemness, and glutamine metabolism by YY1/SLC1A5 pathway
Source: Transl Neurosci. 2024 Feb 24;15(1):20220333. doi: 10.1515/tnsci-2022-0333 (PMC10896183; doi:10.1515/tnsci-2022-0333)
Supplement: Supplementary Figure [file tnsci-2022-0333-sm.pdf]

Supplementary material

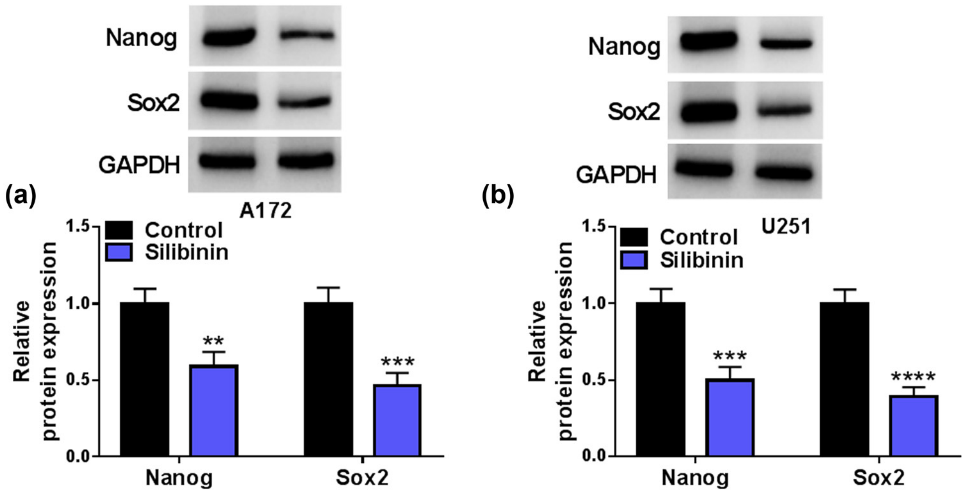

Figure S1: Effect of Silibinin on the protein expression of stemness markers. WB was used to measure the protein levels of Nanog and Sox2 in A172 cells (a) and U251 cells (b) treated with or without Silibinin. **P<0.01, ***P<0.001, ****P<0.0001.

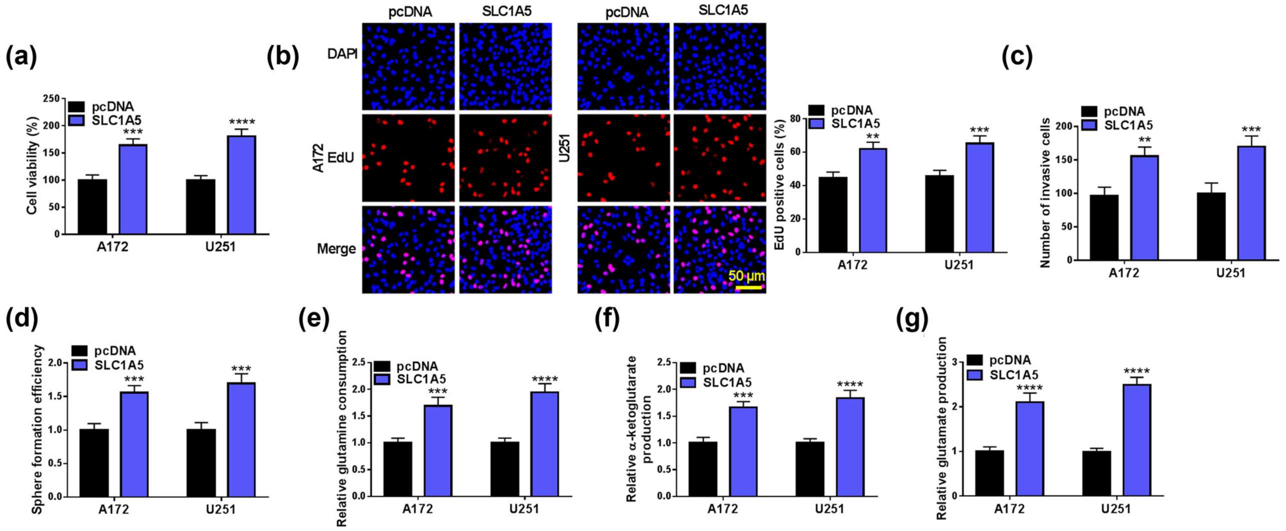

Figure S2: Effect of SLC1A5 on GBM cell progression. A172 and U251 cells were transfected with pcDNA/SLC1A5 overexpression vector. CCK8 assay (a), EdU assay (b), transwell assay (c) and sphere formation assay (d) were used to test cell proliferation, invasion and stemness. (e–g) Glutamine metabolism was assessed by testing glutamine consumption, glutamate production and α-ketoglutarate production. **P<0.01, ***P<0.001, ****P<0.0001.
